# Regional variation in non-traumatic major lower limb amputation in England: observational study of linked primary and secondary care data

**DOI:** 10.1093/bjsopen/zraf004

**Published:** 2025-03-26

**Authors:** Anna Meffen, Mark J Rutherford, Rob D Sayers, John S M Houghton, Naomi Bradbury, Laura J Gray

**Affiliations:** Department of Population Health Sciences, University of Leicester, Leicester, UK; National Institute for Health and Care Research Leicester Biomedical Research Centre, University of Leicester, Leicester, UK; Department of Population Health Sciences, University of Leicester, Leicester, UK; National Institute for Health and Care Research Leicester Biomedical Research Centre, University of Leicester, Leicester, UK; Department of Cardiovascular Sciences, University of Leicester, Leicester, UK; Department of Cardiovascular Sciences, University of Leicester, Leicester, UK; Department of Population Health Sciences, University of Leicester, Leicester, UK; National Institute for Health and Care Research Leicester Biomedical Research Centre, University of Leicester, Leicester, UK; Department of Population Health Sciences, University of Leicester, Leicester, UK; National Institute for Health and Care Research Leicester Biomedical Research Centre, University of Leicester, Leicester, UK

## Abstract

**Background:**

Peripheral artery disease and diabetes are the main primary risk factors for non-traumatic major lower limb amputation. Regional variation in incidence of major lower limb amputation has yet to be fully described in terms of these risk factors and explained. The aim of this study was to estimate yearly incidence of major lower limb amputation over a 10-year interval (2010–2019) across England, by related condition and by region and, additionally, to investigate reasons for regional variation.

**Methods:**

This observational study utilized primary care (Clinical Practice Research Datalink Aurum), secondary care (Hospital Episode Statistics), death and demographic data in England. Adults registered with a practice using Clinical Practice Research Datalink Aurum and with Hospital Episode Statistics linkage were included. Patients with a record of major lower limb amputation during the interval 1 January 2010 to 31 December 2019 were identified and yearly incidence rates of major lower limb amputation were calculated. Co-morbidities analysed were cardiovascular disease (including coronary artery disease, peripheral artery disease and cerebrovascular disease), diabetes (of any type) and cancer. Demographic and socioeconomic covariates analysed were age, sex, ethnicity, deprivation level, region and urban/rural categorization.

**Results:**

The study included 18 397 483 individuals, 8584 of which had a record of major lower limb amputation. The age-standardized yearly incidence rate of major lower limb amputation in England decreased by 30% from 11.2 per 100 000 person-years in 2010 to 7.8 in 2019. The incidence rate in those with diabetes fell by 30% over the 10-year interval, rose by 20% for those with both diabetes and cardiovascular disease, and changed little in those with cardiovascular disease. In 2019, the age-standardized incidence rate was highest in the North East (14.8 per 100 000 person-years) and lowest in the East of England (4.5 per 100 000 person-years). Between 2010 and 1019, incidence rates decreased across all regions, the largest decrease of 56% in the East Midlands and the smallest of 8% in the North East. Statistically significant regional variation remained after full adjustment for demographic, socioeconomic data and related conditions.

**Conclusion:**

Whilst the incidence of major lower limb amputation is decreasing overall, significant regional variation in major lower limb amputation exists and is unexplained by demographic, socioeconomic and health data. Regional differences in service provision and accessibility should be investigated to provide further explanation.

## Teaser

This study estimated the yearly incidence of major lower limb amputation in England over 10 years, overall, by conditions and by regions, while exploring reasons for regional variations. Overall incidence fell by 30%, with similar declines for those with diabetes, but a 20% increase was observed for individuals with both diabetes and cardiovascular disease. Significant regional variation was found, being highest in the North East and lowest in the East of England, remaining after adjustment for demographic, socioeconomic data and related conditions, thus indicating the need to investigate differences in service provision and accessibility.

## Introduction

Peripheral artery disease (PAD) and diabetes are the predominant causes of non-traumatic major lower limb amputation (MLLA)^1^. While improvements in the management of chronic limb-threatening ischaemia (CLTI; the end-stage of PAD) have improved outcomes for those presenting to vascular surgery with potentially salvageable legs, reducing PAD and diabetes-related major amputations requires whole-system improvements in healthcare and close collaboration of primary and secondary care^2^. The epidemiology of MLLA is therefore an important public health measure and regional differences in incidence of amputation need to be understood to achieve ongoing improvements and reduce inequalities of healthcare. However, incidence trends of MLLA in England are widely debated^3–11^. Studies agree that there is regional variation in MLLA incidence, with incidence higher in the north^3,6,9–11^. The reasons for this variation are unexplained by deprivation, age or sex differences. These studies suggest that regional differences in both ethnicity and prevalence of related conditions may be driving regional variation^11^. A systematic review identified that studies either reported for those with PAD or diabetes; no studies reported for or compared MLLA incidence between both^11^.

PAD is a manifestation of systemic cardiovascular disease, and coprevalence of PAD with other cardiovascular diseases, such as coronary artery disease and cerebrovascular disease, is common^12,13^. Accuracy in the recording of PAD diagnoses within general practice (GP) data is debated and PAD is also widely underdiagnosed^14,15^. This renders the process of accurately identifying those with PAD in electronic health record studies difficult. Therefore, in order to capture as many individuals at risk of MLLA due to PAD in the population as possible, cardiovascular disease (CVD) can be used as a proxy for PAD.

Studies to date have used the secondary care database Hospital Episode Statistics (HES) to ascertain MLLA data and separate aggregate data sources, such as national health survey and census data, as population data^3,9,16,17^. As it is event based, HES does not contain general population data. Using aggregate population data, which differs in source from the case data, results in differing inclusion criteria between the case and population data, introducing bias into incidence calculations and leading to inaccurate results. Furthermore, these aggregate data are only stratified by a minimal number of subgroups, often lacking health-related data. A more accurate way to assess if the regional morbidity rate differences affect MLLA incidence is to ascertain patient-level case and population morbidity rate information from primary care health records.

This study aimed to describe time trends in non-traumatic MLLA across England between 2010 and 2019, by morbidity rate (CVD and/or diabetes) and by region using linked secondary and primary care data, and also to investigate reasons for regional variation.

## Methods

A population-based observational study was performed using the population contained within the primary care database of Clinical Practice Research Datalink (CPRD) Aurum (CPRD registration number 21_000364, approved on 17 March 2021).

### Inclusion/exclusion criteria

Adults were included if they were registered within a CPRD Aurum practice that was considered by CPRD to be ‘registered acceptable’ with ‘up-to-standard follow-up’ and had available HES linkage.

MLLA was defined as a lower limb amputation above the ankle. Individuals were defined as having an incident MLLA if they had an Office of Population Censuses and Surveys Classification of Surgical Operations and Procedures (4th revision) (OPCS-4) code for MLLA recorded in the HES linkage within the study interval of 1 January 2010 to 31 December 2019 inclusive. The recording of MLLA in primary care data may be inaccurate^18^. MLLA were excluded where a code for traumatic injury was present within the same hospital episode. Should an individual be recorded as having more than one MLLA, either on the same or a different limb, then only the amputation at the highest level was included in analysis to reflect greatest severity. Where bilateral amputations at the same level were recorded, only the first recorded was included to avoid double counting.

The denominator data set was created to match the criteria for that of the case data set minus criteria related to MLLA.

CPRD limitations on health data access meant that, whilst it was possible to use CPRD demographic data for the whole-study population, access to health data was only granted for individuals with a case of MLLA and for a large random sample of the CPRD population. Where analysis involved health data, the sample data were used and upweighted to represent the population. Where health data were not used in the analysis, the above defined population data were used.

### Covariates

Demographic and socioeconomic covariates analysed were age, sex, ethnicity, deprivation level, region and urban/rural categorization, chosen based on availability of data and potential to explain regional variation suggested by a systematic review^11^. Age was grouped as: below 50, 50–59, 60–69, 70–79 and 80 years and over. Ethnicity categories were defined by the Office of National Statistics (ONS) including a category for missing ethnicity^19^. Ethnicity data was taken from HES records then CPRD where missing in HES^20^. Patient-level Index of Multiple Deprivation 2015 quintiles were used to categorize deprivation levels^21^. Region was defined as patient-level Strategic Health Authority (SHA) regions. Urban/rural 2011 classification data were obtained at practice level due to restrictions by CPRD. A ‘missing’ category was created for all categorical variables where missingness was found; individuals were excluded who had missing region data, however, missingness of region was negligible.

As accuracy in the recording of PAD diagnoses within GP data is debated and PAD underdiagnosed; accurately identifying those with PAD in electronic health record studies is difficult^14,15^. CVD was used as a proxy in order to capture as many patients at risk as accurately as possible. CVD included coronary artery disease, PAD and cerebrovascular disease codes. Subsequently, co-morbidities analysed were CVD, diabetes (of any type) and cancer. Individuals were included as having these co-morbidities on the first instance of a relevant code. Limb amputation due to cancer is rare, consequently, the inclusion of cancer-related amputations is unlikely to impact results. However, cancer and CVD share many risk factors and a history of cancer can increase an individual’s risk of CVD^22^. Amputations of those with a history of cancer were therefore not excluded; cancer was included in the analysis as a variable that may increase risk/explain regional variation^23^.

Condition diagnoses are defined using the medical code lists that are available online at: https://github.com/AnnaMeffen/MLEA_incidence_thesis_supplements.

### Analysis methods

Covariates were summarized by data set, case, sample population and whole CPRD population (where data were available) and presented as mean(s.d.) for continuous variables and count (%) for categorical variables.

Crude and age-standardized incidence rates were calculated yearly for the whole CPRD population and by region. As diagnoses data were not available for the whole CPRD population, a sample and upweighting method was used to calculate the yearly incidence rate stratified by CVD and diabetes (*Supplementary methods*).

Person-years at risk were defined as the difference between the entry and exit date divided by 365.25. Entry date was defined as the latest of 1 January of the year the person turned 18 years old, study start date and registration date. Exit date was defined as the earliest of study end date, date of death and registration end date. Study start and end dates were defined calendar yearly for the whole 10-year study interval.

Death date was taken from ONS and then from CPRD where ONS death data was not recorded. CPRD provides death data, however, ONS is believed to be more accurate^24^.

The incidence rate was defined as the total number of new events out of the total person-years at risk and presented per 100 000 person-years. Age-standardized incidence rates were calculated using the age group cut-points defined above with rates standardized to the age distribution of the whole CPRD denominator data set in 2010. Upweighted and age-standardized incidence rates were stratified by morbidity rate: those with diabetes, those with CVD, and individuals with both diabetes and CVD (these groups are not mutually exclusive).

Factors influencing regional variation were investigated by applying negative binomial models to the upweighted data sets for the whole 10-year study interval then varying the included covariates. Initially, an unadjusted model containing region as the only covariate, based on the crude regional incidence estimates, was applied to provide comparison and assess crude levels of variation. As upweighting was performed using region, sex and age group for each study year, these covariates were added to form the adjusted base model. The remaining covariates were added individually to the adjusted base model and then together as a fully adjusted model. The incidence rate ratios (IRRs) of these models were compared by region, with London (the most populous region) as the base comparator.

Analyses were performed using Stata v. 18^25^, R 4.3.1^26^ and RStudio 2023.03.0^27^.

## Results

### Case data set demographic

There were 8584 patients who underwent at least one MLLA within the time interval identified (*Table 1*). The mean age at event was 70 years with over two-thirds being male (67%), the majority were of white ethnicity (89%) and most lived in an urban setting (87%). CVD was more prevalent than diabetes (58% *versus* 53%) and about a fifth of patients had a diagnosis of cancer. Deprivation quintiles were not evenly distributed, with 90% more MLLA in the most deprived quintile than in the least deprived quintile. The highest number of MLLA was seen in the North West of England with the lowest in the East Midlands.

**Table 1:** Patient demographics by data set

Demographic	MLLA patients	Sample population	CPRD population
Age (years), mean(s.d.)	70(20)	46(29)	46(29)
Age at death (years), mean(s.d.)	76(17)	82(17)	82(17)
**Sex**			
Male	5768 (67.19)	441 489 (48.47)	8 915 486 (48.46)
Female	2816 (32.81)	469 373 (51.53)	9 481 652 (51.54)
** **Undetermined	0 (0.00)	24 (<0.01)	345 (<0.01)
**Ethnicity**			
** **White	7679 (89.46)	571 053 (62.69)	–
** **Black	236 (2.75)	35 559 (3.90)	–
Asian	153 (1.78)	69 361 (7.61)	–
Other	55 (0.64)	21 101 (2.32)	–
Mixed	38 (0.44)	18 622 (2.04)	–
** **Missing	423 (4.93)	195 190 (21.43)	–
**Region**			
North West	1925 (22.43)	146 008 (16.03)	2 946 618 (16.02)
West Midlands	1470 (17.12)	135 221 (14.84)	2 737 123 (14.88)
South West	1193 (13.90)	113 263 (12.43)	2 277 777 (12.38)
London	954 (11.11)	199 671 (21.92)	4 040 209 (21.96)
South central	932 (10.86)	112 604 (12.36)	2 258 663 (12.28)
South East coast	710 (8.27)	74 243 (8.15)	1 504 187 (8.18)
North East	461 (5.37)	28 326 (3.11)	576 047 (3.13)
Yorkshire	379 (4.42)	35 522 (3.90)	719 842 (3.91)
East of England	352 (4.10)	38 381 (4.21)	777 045 (4.22)
** **East Midlands	208 (2.40)	27 647 (3.04)	559 972 (3.04)
**Deprivation level**			
1 (least deprived)	1227 (14.29)	175 281 (19.24)	–
2	1451 (16.90)	179 641 (19.72)	–
3	1658 (19.32)	175 420 (19.26)	–
4	1896 (22.09)	199 013 (21.85)	–
** **5 (most deprived)	2340 (27.26)	179 259 (19.68)	–
Missing	12 (0.14)	2272 (0.25)	–
**Urban/rural classification**			
Urban	7476 (87.09)	810 251 (88.95)	–
** **Rural	1108 (12.91)	100 635 (11.05)	–
Diabetes type 1	815 (9.49)	5579 (0.61)	–
Diabetes type 2	3665 (42.70)	57 295 (6.29)	–
Diabetes—other	31 (0.36)	4889 (0.54)	–
CVD	4940 (57.55)	66 520 (7.30)	–
Cancer	1725 (20.10)	74 199 (8.15)	–
Total	8584	910 886	18 397 483

Values are *n* (%) unless otherwise specified. Age is at event for MLLA patients and at mid-study for denominator populations. MLLA, major lower limb amputation; CPRD, Clinical Practice Research Datalink; CVD, cardiovascular disease.

### Denominator data sets demographic

In terms of age, sex and region, the sample data set was comparable to that of the whole CPRD data set. Whilst it is not possible to compare other demographics between the sample and whole CPRD populations the proportions of those with diabetes and CVD are similar to those cited in the UK population^28,29^.

### Incidence rate of MLLA in England

**Fig. 1:**
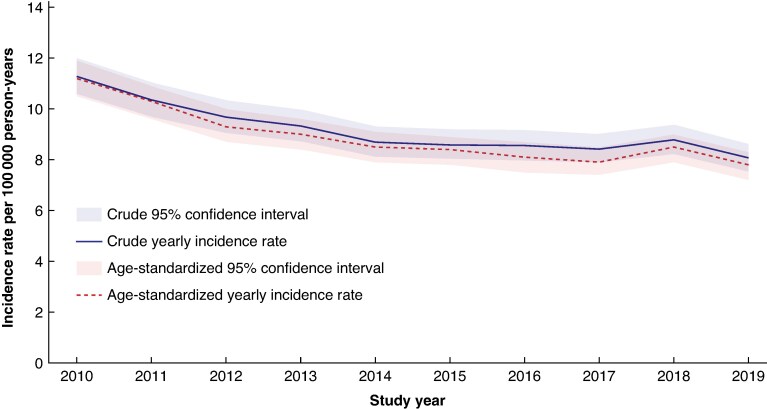
Crude and age-standardized yearly incidence rates of MLLA in England per 100 000 person-years 2010–2019

The crude yearly incidence rate of MLLA in England decreased by 28% over the 10-year study interval whilst the age-standardized yearly incidence rate decreased by 30%. The crude incidence rate fell from 11.3 per 100 000 person-years in 2010 (95% c.i. 10.6 to 12.0) to 8.1 per 100 000 person-years in 2019 (95% c.i. 7.5 to 8.6) and the age-standardized yearly incidence rate fell from 11.2 per 100 000 person-years in 2010 (95% c.i. 10.5 to 11.9) to 7.8 per 100 000 person-years in 2019 (95% c.i. 7.2 to 8.3). The effect of age-standardization was a slightly lower incidence rate overall. The precision of incidence rate estimates remained similar (*Fig. 1*).

Age-standardized to the age distribution of the CPRD study population in mid-2010 categorized as aged less than 50, 50–59, 60–69, 70–79 and 80 years and over. CPRD, Clinical Practice Research Datalink.

### Incidence rate of MLLA by morbidity rate

**Fig. 2:**
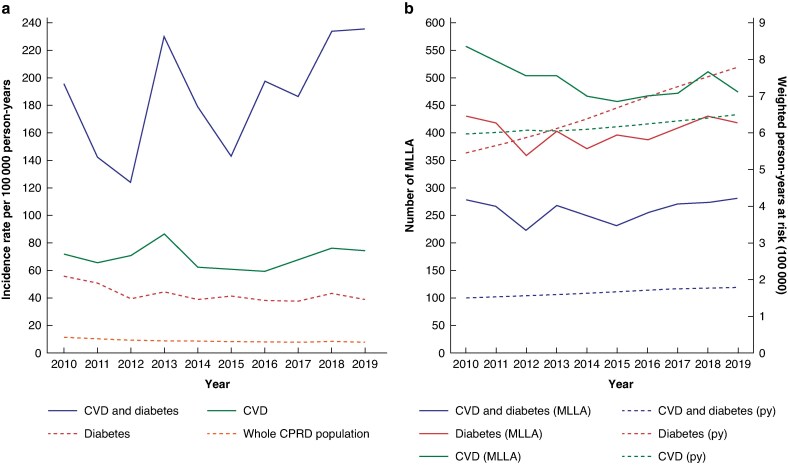
a Yearly incidence rate of MLLA and b number of MLLA and number of person-years at risk by morbidity rate

The age-standardized incidence rate of MLLA in those with diabetes fell by 30% over the 10-year interval (55.5 to 39 per 100 000 person-years) despite an increase in person-years at risk within the study population over the study interval (*Fig. 2*). The incidence rate rose slightly by 3% (71.9 to 74.3 per 100 000 person-years) in those with CVD despite a slight rise in person-years at risk within the study interval. There was a moderate overall increase in the incidence rate of those with both diabetes and CVD over the 10-year study interval of 20% (195.8 to 235.6 per 100 000 person-years), however, there was less stability in incidence rates over time for this group with clear fluctuations.

Uses sample data upweighted to the CPRD study population in mid-2010 in terms of age (categorized as aged less than 50, 50–59, 60–69, 70–79 and 80 years and over), sex and region. Age-standardized to the age distribution of the CPRD study population in mid-2010 categorized as aged less than 50, 50–59, 60–69, 70–79 and 80 years and over. CVD, cardiovascular disease; MLLA, major lower limb amputation; py, person years; CPRD, Clinical Practice Research Datalink.

Incidence rates in those with both CVD and diabetes were highest and were up to 30 times higher than that of the whole population (2019), up to six times higher than those with diabetes and up to three times higher than those with CVD (2019).

### Regional incidence of MLLA

**Fig. 3:**
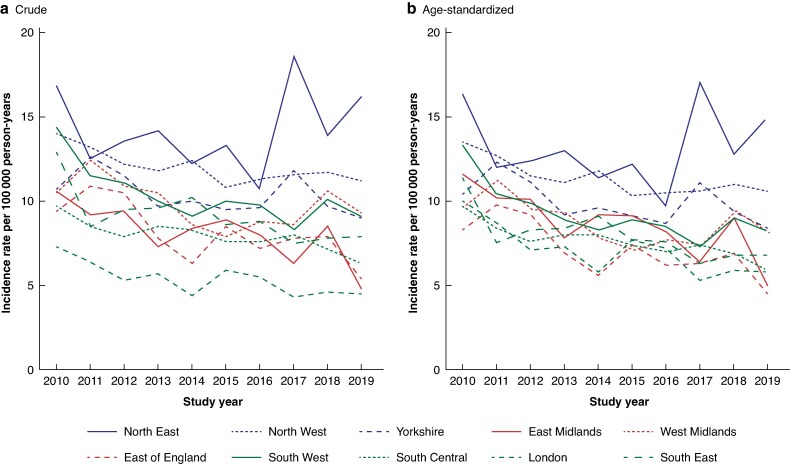
a Crude *versus* b age-standardized incidence rate by region and year

At the start of the study interval, crude and age-standardized incidence rates were highest in the North East (crude: 16.9 per 100 000 person-years, age-standardized: 16.3 per 100 000 person-years with 95% c.i. (11.9 to 20.7)); crude incidence was lowest in London (7.3 per 100 000 person-years) and age-standardized incidence was lowest in the East of England (8.3 per 100 000 person years 2010 with 95% c.i. (5.7 to 11.0)). All regions experienced a decrease in age-standardized incidence rates from 2010 to 2019. The largest decrease of 57% was seen in the East Midlands and the smallest of 8% in the North East (*Fig. 3*). Despite the overall decrease in incidence over the study interval, there was instability in incidence rates with peaks in incidence rates above the initial 2010 rate in some areas.

Age-standardized to the age distribution of the CPRD study population in mid-2010 categorized as aged less than 50, 50–59, 60–69, 70–79 and 80 years and over. Regions are grouped into north, Midlands and south by colour. CPRD, Clinical Practice Research Datalink.

The effect of age-standardization was a reduction in the magnitude of regional variation (*Fig. 3*), suggesting that differences in age between regions plays a small part in regional variation.

Looking at regions grouped into north, Midlands and south (*Fig. 3*), the incidence of MLLA was higher in northern regions, with little difference between the Midlands and southern regions.

An interactive R shiny app has been created to visualize crude and age-standardized incidence rates of MLLA in map format and is available at: https://annameffen.shinyapps.io/MLLA_by_SHA/.

### Reasons for regional variation

**Fig. 4:**
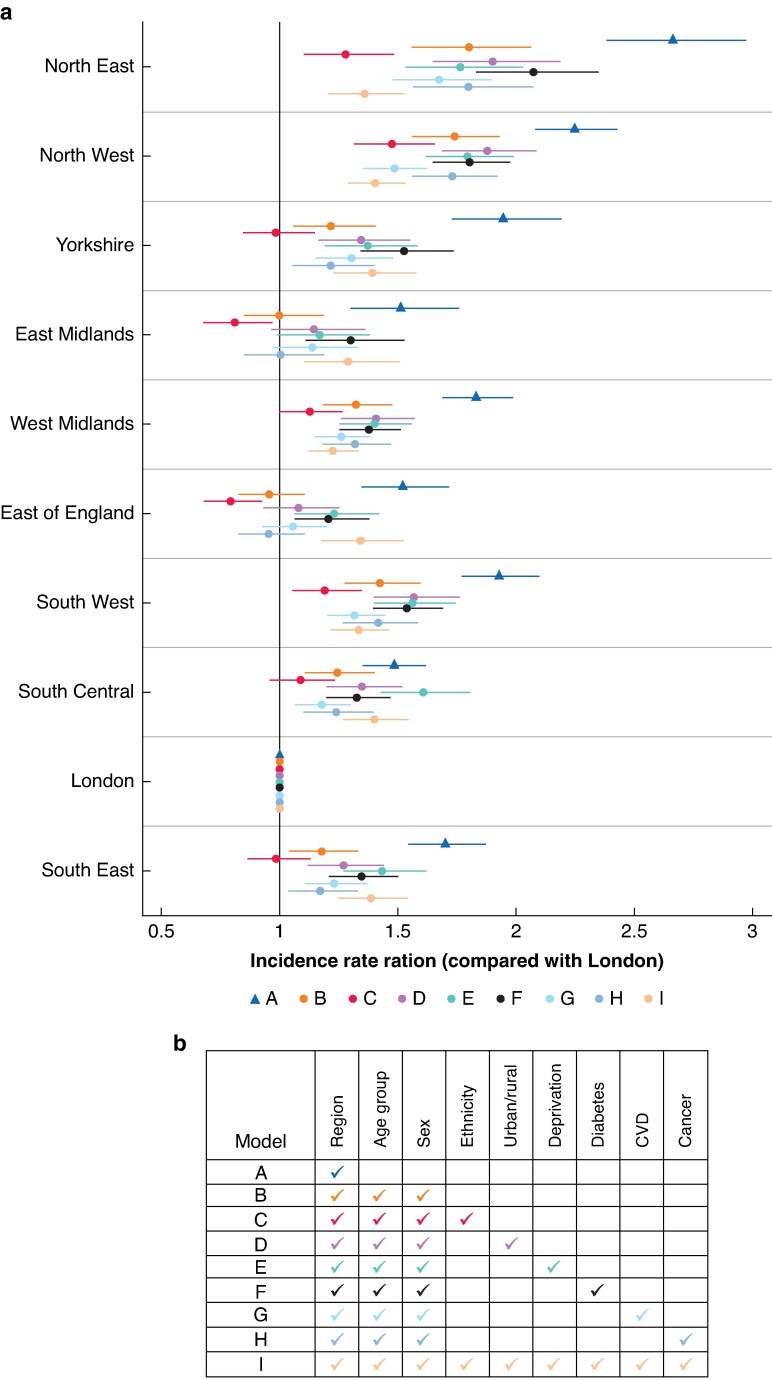
a Incidence rate ratios and confidence interval of each model applied by region compared with London over the whole 10-year study interval b Model definitions

Over the 10-year interval, crude incidence was significantly higher than London in all regions and highest in the North East (IRR 2.66, 95% c.i. 2.37 to 2.98) (*Fig. 4*). Age and sex (model B) explained some of the variation in MLLA incidence compared with London, particularly in the East Midlands and the East of England where the incidence rate was not significantly different from London after age and sex adjustment. Variation in MLLA incidence rates between London and other regions was most often explained by differences in ethnicity compared with London (model C). After adjustment for age, sex and ethnicity, incidence rates were not significantly different from London in Yorkshire, south central and the South East; incidence rates were significantly lower than London in the East Midlands (IRR 0.81, 95% c.i. 0.68 to 0.97) and the East of England (IRR 0.79, 95% c.i. 0.68 to 0.93). Differences in proportions of deprivation (model E) and proportions of those with diabetes (model F) or cancer (model I) played little part in variation according to these models. Regional variation was not explained by any of the models in the North East, North West and South West, where incidence rates were highest. When fitting the fully adjusted model (model I), MLLA incidence remained significantly different from London in all regions.

Model A uses the full study data and is based on crude incidence rates. All remaining models (models B through to I) use sample data upweighted to the CPRD study population in each mid-year in terms of age (categorized as aged less than 50, 50–59, 60–69, 70–79 and 80 years and over), sex and region. Model B, including age, sex and region, acts as a base model given the data was upweighted using these variables. CPRD, Clinical Practice Research Datalink; CVD, cardiovascular disease.

## Discussion

While the overall incidence of MLLA in England is declining, there are significant regional variations that are partially influenced by age and ethnicity. However, differences in deprivation, diabetes or cancer prevalence did not account for this. This suggests that variation in design and delivery of healthcare services may be contributing to the differing MLLA incidence rates across regions.

Comparison to previous studies is challenging due to methodological differences^11^. While many studies focused on diabetes-related amputations^7,8,10,11,30–38^, only two examined CVD-related amputations (PAD specific) each with varying data sets and criteria^5,9,11^. None compared amputation incidence between CVD and diabetes or reported rates for the general population in a similar interval. One diabetes-focused study reported an incidence rate but their narrower criteria (type 2 diabetes only) and the lack of standardization may explain their higher rate of 96 per 100 000 person-years.

Despite methodological differences, similar conclusions were reached in studies by Holman^10^, Ahmad^3^, Moxey^6^ and Maheswaran^9^, showing higher amputation incidence in the north and lower rates in regions that are more ethnically diverse, as seen here in London and the East Midlands, though not in the West Midlands. While previous studies suggested that regional variation might reflect varying prevalence of related conditions, this study found no evidence to support that. Additionally, these studies suggest that regional variation may stem from regional differences in healthcare accessibility and service provision.

It is encouraging that MLLA incidence rates steadily declined for those with diabetes despite diabetes prevalence increasing over the same interval^39^. This has coincided with national efforts to improve and standardize diabetic footcare. The England and Wales National Diabetic Foot Audit and Quality Improvement Collaborative has demonstrated regional variation in terms of service delivery and outcomes^40^. They recommend increasing accessibility of, and time to assessment by specialist, multidisciplinary foot services^40^. However, it is notable that, in this study, the majority of MLLAs were performed in those with CVD and no such decrease in MLLA was observed in this cohort. PAD is often recognized late and potential missed opportunities in timely specialist referrals have been identified, with delays to specialist vascular assessment likely increasing the risk of MLLA^41,42^. Rapid-access specialist limb-salvage clinics have demonstrated reduction in MLLA incidence^43–45^. UK guidelines now specify time targets from referral to revascularization of those with CLTI and additional National Health Service (NHS) funding to achieve them, which may reduce some regional variation^46^. However, much of the regional variation may exist upstream of specialist vascular clinics^47,48^. It is notable that few PAD guidelines (including guidelines on referral) included primary care clinicians and it is vital future guidelines include all key stakeholders in their author group^49^.

This was both the first study to compare incidence of MLLA in England by morbidity rate over time, and the first to assess time trends and reasons for regional variation using primary care data. More specifically, this study is the first to explore regional differences in morbidity rate as a reason for regional variation. Importantly, the numerator and denominator data were derived from the same population data source, minimizing potential bias within the results. This study includes detailed descriptions of methodology, precisely defined statistical terminology and adhered to recommended reporting guidelines (*Table S1*).

The primary limitation of this study is that CPRD does not cover the entire population of England, meaning this study is more akin to a cohort study. The study population size is further restricted by CPRD data size access limitations leading to the necessity of the upweighting process. Population coverage limitations are balanced by the accuracy gained from capturing both numerator and denominator data from the same population, as discussed earlier. Research has shown CPRD data to be representative in terms of age, sex and ethnicity for the whole population, however, this is not confirmed regionally^50,51^. Clinical representativeness in terms of morbidity rate prevalence is not described in available literature. CPRD data is also likely to be biased towards a less healthy population as healthy people may not register at a GP surgery and be represented. Additionally, these data may contain duplicate records where individuals have changed GP practices, thus contributing to the CPRD database under multiple patient identifiers^52^.

On stratification, there was little MLLA patient data for non-white ethnicities, which could be reflective of the population and the nature of the causes of MLLA (which is most likely), but there remains a small risk that it could be reflective of bias due to unreliable data collection methods; it is not possible to confirm with the available data^53,54^. As any missing data in MLLA patients with non-white ethnicity could potentially make a small, but likely insignificant, difference to results, caution is advised to not overstate the role of ethnicity. Further limitations may lie in the accuracy of diagnostic coding. Whilst care has been taken, there are risks of inaccuracies in both the code lists and in recording of all collected data, though the risks are likely to be small with little impact on results.

Significant regional variation in MLLA incidence in England exists after adjustment for patient demographic, socioeconomic factors and related conditions. Routinely collected electronic health records are unlikely to provide further insight into the remaining variation; regional differences in service provision and accessibility should be investigated to provide further explanation and determine where healthcare system redesign may reduce regional disparity in MLLA incidence.

## Supplementary Material

zraf004_Supplementary_Data

## Data Availability

Data are not publicly available. Data access via CPRD is subject to protocol approval via CPRD’s Research Data Governance (RDG) Process.

## References

[1] Centers for Diseases and Control Prevention . Preventing Diabetes-Related Amputations. 2023. https://www.cdc.gov/diabetes/diabetes-complications/preventing-diabetes-related-amputations.html (accessed 31 October 2024)

[2] Conte MS, Bradbury AW, Kolh P, White JV, Dick F, Fitridge R et al Global vascular guidelines on the management of chronic limb-threatening ischemia. Eur J Vasc Endovasc Surg 2019;58:S1–S109.e33 31182334 10.1016/j.ejvs.2019.05.006 PMC8369495

[3] Ahmad N, Thomas GN, Gill P, Chan C, Torella F. Lower limb amputation in England: prevalence, regional variation and relationship with revascularisation, deprivation and risk factors. A retrospective review of hospital data. J R Soc Med 2014;107:483–489 25389229 10.1177/0141076814557301 PMC4265106

[4] Ahmad N, Thomas GN, Gill P, Torella F. The prevalence of major lower limb amputation in the diabetic and non-diabetic population of England 2003–2013. Diab Vasc Dis Res 2016;13:348–353 27334482 10.1177/1479164116651390

[5] Behrendt CA, Sigvant B, Szeberin Z, Beiles B, Eldrup N, Thomson IA et al International variations in amputation practice: a VASCUNET report. Eur J Vasc Endovasc Surg 2018;56:391–399 29859821 10.1016/j.ejvs.2018.04.017

[6] Moxey PW, Hofman D, Hinchliffe RJ, Jones K, Thompson MM, Holt PJ. Epidemiological study of lower limb amputation in England between 2003 and 2008. Br J Surg 2010;97:1348–1353 20632310 10.1002/bjs.7092

[7] Vamos EP, Bottle A, Edmonds ME, Valabhji J, Majeed A, Millett C. Changes in the incidence of lower extremity amputations in individuals with and without diabetes in England between 2004 and 2008. Diabetes Care 2010;33:2592–2597 20833865 10.2337/dc10-0989 PMC2992196

[8] Vamos EP, Bottle A, Majeed A, Millett C. Trends in lower extremity amputations in people with and without diabetes in England, 1996–2005. Diabetes Res Clin Pract 2010;87:275–282 20022126 10.1016/j.diabres.2009.11.016

[9] Maheswaran R, Tong T, Michaels J, Brindley P, Walters S, Nawaz S. Time trends and geographical variation in major lower limb amputation related to peripheral arterial disease in England. BJS Open 2024;8:zrad140 38180913 10.1093/bjsopen/zrad140 PMC10768980

[10] Holman N, Young RJ, Jeffcoate WJ. Variation in the recorded incidence of amputation of the lower limb in England. Diabetologia 2012;55:1919–1925 22398645 10.1007/s00125-012-2468-6

[11] Meffen A, Houghton JSM, Nickinson ATO, Pepper CJ, Sayers RD, Gray LJ. Understanding variations in reported epidemiology of major lower extremity amputation in the UK: a systematic review. BMJ Open 2021;11:e053599 10.1136/bmjopen-2021-053599 PMC8496376 34615685

[12] Criqui MH, Aboyans V. Epidemiology of peripheral artery disease. Circ Res 2015;116:1509–1526 25908725 10.1161/CIRCRESAHA.116.303849

[13] Jensen PS, Petersen J, Kirketerp-Møller K, Poulsen I, Andersen O. Progression of disease preceding lower extremity amputation in Denmark: a longitudinal registry study of diagnoses, use of medication and healthcare services 14 years prior to amputation. BMJ Open 2017;7:e016030 10.1136/bmjopen-2017-016030 PMC5695421 29101132

[14] Behroozian AA, Beckman JA. Asymptomatic peripheral artery disease: silent but deadly. Prog Cardiovasc Dis 2021;65:2–8 33617896 10.1016/j.pcad.2021.02.009 PMC11824944

[15] Kyle D, Boylan L, Wilson L, Haining S, Oates C, Sims A et al Accuracy of peripheral arterial disease registers in UK general practice: case-control study. J Prim Care Community Health 2020;11:2150132720946148 32959726 10.1177/2150132720946148 PMC7513392

[16] NHS Digital . Health survey for England 2023. https://digital.nhs.uk/data-and-information/publications/statistical/health-survey-for-england (accessed 31 October 2024)

[17] Office for National Statistics . Census. 2024. https://www.ons.gov.uk/census (accessed 31 October 2024)

[18] Meffen A, Sayers RD, Gillies CL, Khunti K, Gray LJ. Are major lower extremity amputations well recorded in primary care electronic health records?: insights from primary care electronic health records in England. Prim Health Care Res Dev 2022;23:e77 36440656 10.1017/S1463423622000718 PMC9706375

[19] Office for National Statistics . Ethnicity and race. 2023. https://service-manual.ons.gov.uk/content/language/ethnicity-and-race (accessed 31 October 2024)

[20] Shiekh SI, Harley M, Ghosh RE, Ashworth M, Myles P, Booth HP et al Completeness, agreement, and representativeness of ethnicity recording in the United Kingdom’s clinical practice research datalink (CPRD) and linked hospital episode statistics (HES). Popul Health Metr 2023;21:3 36918866 10.1186/s12963-023-00302-0 PMC10013294

[21] GOV.UK . English indices of deprivation 2015. 2023. https://www.gov.uk/government/statistics/english-indices-of-deprivation-2015 (accessed 31 October 2024)

[22] Raisi-Estabragh Z, Cooper J, McCracken C, Crosbie EJ, Walter FM, Manisty CH et al Incident cardiovascular events and imaging phenotypes in UK Biobank participants with past cancer. Heart 2023;109:1007–1015 37072241 10.1136/heartjnl-2022-321888 PMC10314020

[23] Arık A, Dodd E, Streftaris G. Cancer morbidity trends and regional differences in England—a Bayesian analysis. PLoS One 2020;15:e0232844 32433663 10.1371/journal.pone.0232844 PMC7239391

[24] Gallagher AM, Dedman D, Padmanabhan S, Leufkens HGM, de Vries F. The accuracy of date of death recording in the clinical practice research datalink GOLD database in England compared with the Office for National Statistics death registrations. Pharmacoepidemiol Drug Saf 2019;28:563–569 30908785 10.1002/pds.4747 PMC6593793

[25] StataCorp. *Stata Statistical Software: Release 18 [software]*. College Station, TX: StataCorp LLC, 2023

[26] R Core Team. *R: A language and environment for statistical computing [software]*. Version 4.3.1. Vienna, Austria: R Foundation for Statistical Computing, 2023

[27] RStudio Team. *RStudio: Integrated Development Environment for R [software]*. Version 2023.03.0. Boston, MA: Posit Software, PBC, 2023

[28] Office for National Statistics . Estimating the number of people with cardiovascular or respiratory conditions living in poverty, England: 2021. 2022. https://www.ons.gov.uk/peoplepopulationandcommunity/healthandsocialcare/healthinequalities/bulletins/estimatingthenumberofpeoplewithcardiovascularorrespiratoryconditionslivinginpovertyengland/2021 (accessed 31 October 2024)

[29] Whicher CA, O’Neill S, Holt RIG. Diabetes in the UK: 2019. Diabet Med 2020;37:242–247 31901175 10.1111/dme.14225

[30] Gunn LH, Vamos EP, Majeed A, Normahani P, Jaffer U, Molina G et al Associations between attainment of incentivized primary care indicators and incident lower limb amputation among those with type 2 diabetes: a population-based historical cohort study. BMJ Open Diabetes Res Care 2021;9:e002069 10.1136/bmjdrc-2020-002069 PMC8076942 33903115

[31] Public Health England . Fingertips public health profiles. 2020. https://fingertips.phe.org.uk/search/amputations#page (accessed 31 October 2024)

[32] Health and Social Care Information Centre . National Diabetes Audit - 2010-11. 2012. https://digital.nhs.uk/data-and-information/publications/statistical/national-diabetes-audit/national-diabetes-audit-2010-11 (accessed 31 October 2024)

[33] Health and Social Care Information Centre . National Diabetes Audit - 2011-12: Report 2. 2013. https://digital.nhs.uk/data-and-information/publications/statistical/national-diabetes-audit/national-diabetes-audit-2011-12-report-2 (accessed 31 October 2024)

[34] Health and Social Care Information Centre . National Diabetes Audit -2012-2013, Report 2. 2015. https://digital.nhs.uk/data-and-information/publications/statistical/national-diabetes-audit/national-diabetes-audit-2012-2013-report-2 (accessed 31 October 2024)

[35] Health and Social Care Information Centre . National Diabetes Audit - Report 2 Complications and Mortality, 2017-18. 2019. https://digital.nhs.uk/data-and-information/publications/statistical/national-diabetes-audit/report-2--complications-and-mortality-2017-18 (accessed 31 October 2024)

[36] Healthcare Quality Improvement Partnership . National Diabetes Audit, 2015-16 Report 2a: Complications and Mortality. 2017. https://files.digital.nhs.uk/pdf/4/t/national_diabetes_audit__2015-16__report_2a.pdf (accessed 31 October 2024)

[37] Kennon B, Leese GP, Cochrane L, Colhoun H, Wild S, Stang D et al Reduced incidence of lower-extremity amputations in people with diabetes in Scotland: a nationwide study. Diabetes Care 2012;35:2588–2590 23011727 10.2337/dc12-0511 PMC3507601

[38] The NHS Information Centre . National Diabetes Audit - 2009-10. 2011. https://digital.nhs.uk/data-and-information/publications/statistical/national-diabetes-audit/national-diabetes-audit-2009-10 (accessed 31 October 2024)

[39] Holman N, Forouhi NG, Goyder E, Wild SH. The association of public health observatories (APHO) diabetes prevalence model: estimates of total diabetes prevalence for England, 2010–2030. Diabet Med 2011;28:575–582 21480968 10.1111/j.1464-5491.2010.03216.x

[40] NHS England . National Diabetes Foot Care Audit 2018 to 2023. 2024. https://digital.nhs.uk/data-and-information/publications/statistical/national-diabetes-footcare-audit/2018-2023# (accessed 31 October 2024)

[41] Nickinson A, Coles B, Payne T, Davies R, Khunti K, Sayers R. Missed opportunities for limb salvage in patient undergoing a major amputation: a cohort study using the clinical practice research datalink. Eur J Vasc Endovasc Surg 2019;58:e569–e570 10.1016/j.ejvs.2020.05.010 32718828

[42] Nickinson ATO, Bridgwood B, Houghton JSM, Nduwayo S, Pepper C, Payne T et al A systematic review investigating the identification, causes, and outcomes of delays in the management of chronic limb-threatening ischemia and diabetic foot ulceration. J Vasc Surg 2020;71:669–81.e2 31676182 10.1016/j.jvs.2019.08.229

[43] Houghton JSM, Meffen A, Gray LJ, Payne TJ, Haunton VJ, Davies RSM et al Streamlined clinical management pathways may reduce major amputations in patients with chronic limb threatening ischaemia: a prospective cohort study with historical controls. Eur J Vasc Endovasc Surg 2024. doi:10.1016/j.ejvs.2024.09.005 39260765

[44] Nickinson ATO, Dimitrova J, Houghton JSM, Rate L, Dubkova S, Lines H et al Does the introduction of a vascular limb salvage service improve one year amputation outcomes for patients with chronic limb-threatening ischaemia? Eur J Vasc Endovasc Surg 2021;61:612–619 33583708 10.1016/j.ejvs.2020.12.007

[45] Nickinson ATO, Houghton JSM, Bridgwood B, Essop-Adam A, Nduwayo S, Payne T et al The utilisation of vascular limb salvage services in the assessment and management of chronic limb-threatening ischaemia and diabetic foot ulceration: a systematic review. Diabetes Metab Res Rev 2020;36:e3326 32314493 10.1002/dmrr.3326

[46] Birmpili P, Atkins E, Boyle JR, Sayers RD, Blacker K, Williams R et al The Vascular PAD-QIF CQUIN: what is it, why is it important, what does it mean for vascular units? J Vasc Soc G B Irel 2022;1:63–64

[47] Atkins E, Birmpili P, Kellar I, Johal AS, Li Q, Waton S et al Understanding delays in chronic limb-threatening ischaemia care: application of the theoretical domains framework to identify factors affecting primary care clinicians’ referral behaviours. J Foot Ankle Res 2024;17:e12015 38703396 10.1002/jfa2.12015 PMC11296715

[48] Atkins E, Kellar I, Birmpili P, Waton S, Li Q, Johal AS et al The symptom to assessment pathway for suspected chronic limb-threatening ischaemia (CLTI) affects quality of care: a process mapping exercise. BMJ Open Qual 2024;13:e002605 10.1136/bmjoq-2023-002605 PMC10824038 38267216

[49] Atkins E, Birmpili P, Kellar I, Glidewell L, Cromwell DA. Documentary analysis of national and international guidance for community clinicians referring patients with suspected chronic limb-threatening ischaemia. BMJ Open Qual 2024;13:e002784 10.1136/bmjoq-2024-002784 PMC11110609 38769026

[50] Herrett E, Gallagher AM, Bhaskaran K, Forbes H, Mathur R, van Staa T et al Data resource profile: clinical practice research datalink (CPRD). Int J Epidemiol 2015;44:827–836 26050254 10.1093/ije/dyv098 PMC4521131

[51] Wolf A, Dedman D, Campbell J, Booth H, Lunn D, Chapman J et al Data resource profile: clinical practice research datalink (CPRD). Int J Epidemiol 2019;48:1740–1740g 30859197 10.1093/ije/dyz034 PMC6929522

[52] NHS Digital . Patients Registered at a GP Practice April 2021. 2021. https://digital.nhs.uk/data-and-information/publications/statistical/patients-registered-at-a-gp-practice/april-2021 (accessed 31 October 2024)

[53] Office for National Statistics (ONS) . Understanding consistency of ethnicity data recorded in health-related administrative datasets in England: 2011 to 2021. 2023. https://www.ons.gov.uk/peoplepopulationandcommunity/healthandsocialcare/healthinequalities/articles/understandingconsistencyofethnicitydatarecordedinhealthrelatedadministrativedatasetsinengland2011to2021/2023-01-16 (accessed 31 October 2024)

[54] Office for National Statistics (ONS) . Methods and systems used to collect ethnicity information in health administrative data sources, England: 2022. 2023. https://www.ons.gov.uk/peoplepopulationandcommunity/healthandsocialcare/healthinequalities/articles/methodsandsystemsusedtocollectethnicityinformationinhealthadministrativedatasourcesengland2022/2023-01-16 (accessed 31 October 2024)

