# Supplementary material for: Regional variation in non-traumatic major lower limb amputation in England: observational study of linked primary and secondary care data
Source: BJS Open. 2025 Mar 26;9(2):zraf004. doi: 10.1093/bjsopen/zraf004 (PMC11938355; doi:10.1093/bjsopen/zraf004)
Supplement: zraf004_Supplementary_Data [file zraf004_supplementary_data.docx]

**Regional variation in non-traumatic major lower limb amputation in England:**

**observational study of linked primary and secondary care data**

Anna Meffen ^a b^, Mark J Rutherford ^a b^, Rob D Sayers ^c^, John S M Houghton ^c^, Naomi Bradbury ^a b^, Laura J Gray ^a b^

^a^ Department of Population Health Sciences, University of Leicester

^b^ National Institute for Health and Care Research Leicester Biomedical Research Centre, University of Leicester

^c^ Department of Cardiovascular Sciences, University of Leicester

**Correspondence to:** Anna Meffen, [anna.meffen@leicester.ac.uk](mailto:anna.meffen@leicester.ac.uk), University of Leicester, Leicester, LE1 7RH, United Kingdom. **ORCID ID: 0000-0002-9491-6110. Twitter: @DrAnnaMeffen**

**Supplementary Materials - Index**

| **Supplementary Methods** | *Page* |
| --- | --- |
| Rationale for upweighting | *2* |
| Methods for upweighting | *2* |
| **Supplementary Guidelines** |  |
| RECORD guidelines | *3-10* |

**Supplementary Methods**

# **Rationale for upweighting**

Clinical Practice Research Datalink (CPRD) have size restrictions on data access under the multi-study licence agreement with University of Leicester. These restrictions are in place as the download, storage and analysis of the entire CPRD (Aurum/GOLD) clinical dataset is beyond the expected capabilities of most institutions. Researchers are given access to a denominator data file containing limited information (year of birth, sex and region) on the whole CPRD population. Clinical and linkage data can only be given on individuals with the investigated outcome(/s), and/or a large random sample of the whole database population.

In this study, clinical and linkage data was accessed for all those fitting the inclusion criteria who had at least one MLEA within the study period and for a large random sample of the CPRD Aurum population to use as a proxy for the whole CPRD Aurum population data.

Given that all case data within the CPRD Aurum cohort were gathered, to apply a relevant population denominator to incidence calculations where clinical and linkage data was involved, the large random sample needed to be upweighted to represent of the size of the whole CPRD Aurum population data. This was achieved in terms of all variables that were available in both the denominator dataset and the sample dataset, namely, age (devised from year of birth), sex region and calendar year. The method for this process is described below.

**Methods for upweighting**

For each study year, distribution of age (group), sex and region at mid-year was calculated in the denominator dataset for individuals included in the study at that point (which includes those with record of MLEA).

The same was then repeated for the sample dataset (this included all those with a record of MLEA and the random sample)

The weight was then calculated from the previous two steps by dividing the distribution (in terms of age, sex, region) for each study year in the whole population denominator dataset by the distribution in the sample dataset.

To apply the weights, the sample data was stratified by age, sex region and year and also by variables that were not available in both datasets (e.g. ethnicity, diabetes diagnosis etc). The aggregated sample dataset including all analysis variables was then upweighted by multiplying the person-year contribution of each combination of variable by the relevant weight (in terms of age, sex, region and year). In doing so, we assume that the age, region, calendar year and sex-specific distribution of cancer, diabetes, CVD, ethnicity, urban/rural classification and deprivation are the same in our large random sample as in the wider CPRD Aurum dataset.

The weighted person-year variable was then used in both the related conditions incidence calculations and in the regional variation modelling.

Note that incidence calculations for related conditions was also age-standardised (after upweighting) using the same methods as detailed in the main manuscript.

**Supplementary Guidelines:**

**The RECORD statement – checklist of items, extended from the STROBE statement, that should be reported in observational studies using routinely collected health data.**

|  | **Item No.** | **STROBE items** | **Location in manuscript where items are reported** | **RECORD items** | **Location in manuscript where items are reported** |
| --- | --- | --- | --- | --- | --- |
| **Title and abstract** | | |  |  |  |
|  | 1 | (a) Indicate the study’s design with a commonly used term in the title or the abstract (b) Provide in the abstract an informative and balanced summary of what was done and what was found | Line 1-60 | RECORD 1.1: The type of data used should be specified in the title or abstract. When possible, the name of the databases used should be included.    RECORD 1.2: If applicable, the geographic region and timeframe within which the study took place should be reported in the title or abstract.    RECORD 1.3: If linkage between databases was conducted for the study, this should be clearly stated in the title or abstract. | Line 1-60 |
| **Introduction** | | |  |  |  |
| Background rationale | 2 | Explain the scientific  background and rationale for the investigation being reported | Lines 61-84 |  | Lines 61-84 |
| Objectives | 3 | State specific objectives, including any prespecified hypotheses | Lines 82-84 |  | Lines 82-84 |
| **Methods** | | |  |  |  |
| Study Design | 4 | Present key elements of study design early in the paper | Lines 86-87 |  | Lines 86-87 |
| Setting | 5 | Describe the setting, locations, and relevant dates, including periods of recruitment, exposure, follow-up, and data collection | Lines 88-106 |  | Lines 88-106 |

| Participants | 6 | 1. *Cohort study* - Give the eligibility criteria, and the sources and methods of selection of participants. Describe methods of follow-up   *Case-control study* - Give the eligibility criteria, and the sources and methods of case ascertainment and control selection. Give the rationale for the choice of cases and controls *Cross-sectional study* - Give the eligibility criteria, and the sources and methods of selection of participants     1. *Cohort study* - For matched studies, give matching criteria and number of exposed and unexposed   *Case-control study* - For matched studies, give matching criteria and the number of controls per case | Lines 88-106 | RECORD 6.1: The methods of study population selection (such as codes or algorithms used to identify subjects) should be listed in detail. If this is not possible, an explanation should be provided.    RECORD 6.2: Any validation studies of the codes or algorithms used to select the population should be referenced. If validation was conducted for this study and not published elsewhere, detailed methods and results should be provided.    RECORD 6.3: If the study involved linkage of databases, consider use of a flow diagram or other graphical display to demonstrate the data linkage process, including the number of individuals with linked data at each stage. | Lines 88-106 |
| --- | --- | --- | --- | --- | --- |
| Variables | 7 | Clearly define all outcomes, exposures, predictors, potential confounders, and effect modifiers. Give diagnostic criteria, if applicable. | Lines 106-129 | RECORD 7.1: A complete list of codes and algorithms used to classify exposures, outcomes, confounders, and effect modifiers should be provided. If these cannot be reported, an explanation should be provided. | Lines 106-129 |
| Data sources/ measurement | 8 | For each variable of interest, give sources of data and details of methods of assessment (measurement).  Describe comparability of assessment methods if there is  more than one group | Lines 88-129 |  | Lines 88-129 |

| Bias | 9 | Describe any efforts to address potential sources of bias | Lines 88-129 |  | Lines 88-129 |
| --- | --- | --- | --- | --- | --- |
| Study size | 10 | Explain how the study size was arrived at | Lines 88-129 |  | Lines 88-129 |
| Quantitative variables | 11 | Explain how quantitative variables were handled in the analyses. If applicable, describe which groupings were chosen,  and why | Lines 106-129 |  | Lines 106-129 |
| Statistical methods | 12 | (a) Describe all statistical methods, including those used to control for confounding (b) Describe any methods used to examine subgroups and interactions   1. Explain how missing data were addressed 2. *Cohort study* - If applicable, explain how loss to follow-up was addressed   *Case-control study* - If applicable, explain how matching of cases and controls was addressed  *Cross-sectional study* - If applicable, describe analytical methods taking account of sampling strategy   1. Describe any sensitivity analyses | Lines 130-159 |  | Lines 130-159 |
| Data access and cleaning methods |  | .. |  | RECORD 12.1: Authors should describe the extent to which the investigators had access to the database population used to create the study population. | Lines 88-159 |

|  |  |  |  | RECORD 12.2: Authors should provide information on the data cleaning methods used in the study. | Lines 88-159 |
| --- | --- | --- | --- | --- | --- |
| Linkage |  | .. |  | RECORD 12.3: State whether the study included person-level,  institutional-level, or other data linkage across two or more databases. The methods of linkage and methods of linkage quality evaluation should be provided. | Lines 88-159 |
| **Results** | | | | | |
| Participants | 13 | 1. Report the numbers of individuals at each stage of the study (*e.g.*, numbers potentially eligible, examined for eligibility, confirmed eligible, included in the study, completing follow-up, and analysed) 2. Give reasons for nonparticipation at each stage. (c) Consider use of a flow diagram | Lines 160-172 | RECORD 13.1: Describe in detail the selection of the persons included in the study (*i.e.,* study population selection) including filtering based on data quality, data availability and linkage. The selection of included persons can be described in the text and/or by means of the study flow diagram. | Lines 160-172 |
| Descriptive data | 14 | 1. Give characteristics of study participants (*e.g.*, demographic, clinical, social) and information on exposures and potential   confounders   1. Indicate the number of participants with missing data for each variable of interest (c) *Cohort study* - summarise follow-up time (*e.g.*, average and total amount) | Lines 160-172 |  |  |
| Outcome data | 15 | *Cohort study* - Report numbers of outcome events or summary measures over time  *Case-control study* - Report numbers in each exposure | Lines 160-172 |  |  |

|  |  | category, or summary measures of exposure  *Cross-sectional study* - Report numbers of outcome events or summary measures |  |  |  |
| --- | --- | --- | --- | --- | --- |
| Main results | 16 | (a) Give unadjusted estimates and, if applicable, confounderadjusted estimates and their precision (e.g., 95% confidence interval). Make clear which confounders were adjusted for and why they were included (b) Report category boundaries when continuous variables were categorized  (c) If relevant, consider translating estimates of relative risk into absolute risk for a meaningful time period | Lines 176-223 |  |  |
| Other analyses | 17 | Report other analyses done—  e.g., analyses of subgroups and interactions, and sensitivity analyses | Lines 184-233 |  |  |
| **Discussion** | | | | | |
| Key results | 18 | Summarise key results with reference to study objectives | Lines 225-229 |  |  |
| Limitations | 19 | Discuss limitations of the study, taking into account sources of potential bias or imprecision. Discuss both direction and magnitude of any potential bias | Lines 270-284 | RECORD 19.1: Discuss the  implications of using data that were not created or collected to answer the specific research question(s). Include discussion of misclassification bias, unmeasured confounding, missing data, and changing eligibility over time, as they pertain to the study being reported. | Lines 270-284 |
| Interpretation | 20 | Give a cautious overall interpretation of results considering objectives, | Lines 224-290 |  |  |
|  |  | limitations, multiplicity of analyses, results from similar studies, and other relevant evidence | Lines 230-245 |  |  |
| Generalisability | 21 | Discuss the generalisability (external validity) of the study results | Lines 224-290 |  |  |
| **Other Information** | | | | | |
| Funding | 22 | Give the source of funding and the role of the funders for the present study and, if applicable, for the original study on which the present article is based | Lines 319-326 |  |  |
| Accessibility of protocol, raw data, and programming code |  | .. | Lines 295-297 | RECORD 22.1: Authors should provide information on how to access any supplemental information such as the study protocol, raw data, or programming code. | Lines 295-297 |

*Reference: Benchimol EI, Smeeth L, Guttmann A, Harron K, Moher D, Petersen I, Sørensen HT, von Elm E, Langan SM, the RECORD Working Committee. The REporting of studies Conducted using Observational Routinely-collected health Data (RECORD) Statement. *PLoS Medicine* 2015; in press.

*Checklist is protected under Creative Commons Attribution ([CC BY)](http://creativecommons.org/licenses/by/4.0/) lice
